# Subsynovial connective tissue development in the rabbit carpal tunnel

**DOI:** 10.1002/vms3.281

**Published:** 2020-05-06

**Authors:** Verena J. M. M. Schrier, Alyssa Vrieze, Peter C. Amadio

**Affiliations:** ^1^ Biomechanics Laboratory and Tendon and Soft Tissue Biology Laboratory Mayo Clinic Rochester MN USA; ^2^ Department of Plastic, Reconstructive and Hand Surgery Erasmus Medical Center Rotterdam the Netherlands

**Keywords:** Animal model, Carpal Tunnel Syndrome, Subsynovial Connective Tissue

## Abstract

The carpal tunnel contains the digital flexor tendons and the median nerve, which are embedded in a unique network of fibrovascular interconnected subsynovial connective tissue (SSCT). Fibrous hypertrophy of the SSCT and subsequent adaptations in mechanical response are found in patients with carpal tunnel syndrome (CTS), but not much is known about the development of the SSCT. This observational study describes the morphological development of SSCT using histology and ultramicroscopy in an animal model at four time points between late‐term fetuses through adulthood. A transition is seen between 3 days and 6 weeks post‐partum from a dense solid SSCT matrix to a complex multilayered structure connected with collagenous fibrils. These preliminary data show a developmental pattern that matches an adaptive response of the SSCT to loading and motion. Understanding the anatomical development aids in recognizing the pathophysiology of CTS and supports research on new therapeutic approaches.

## Graphical abstract

Connective tissue in the carpal tunnel shows specialized structure reorganization in early development in an animal model. The multilayered architecture could be a response to tendon loading and understanding its mechanistic role could aid in understanding carpal tunnel syndrome pathophysiology.

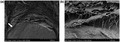

## 1 INTRODUCTION

Carpal tunnel syndrome (CTS) is a very common compression neuropathy at the level of the carpal tunnel (CT), a fibroosseous channel in the wrist. CTS occurs most frequently in adults aged 45–55 (Gelfman et al., 2009), and thus has a substantial socio‐economic impact, with overall costs in the United States alone reaching over $2 billion annually (Palmer & Hanrahan, 1995). Most cases have no directly relatable cause and CTS pathophysiology is still a common topic of investigation.

**Figure 1:**
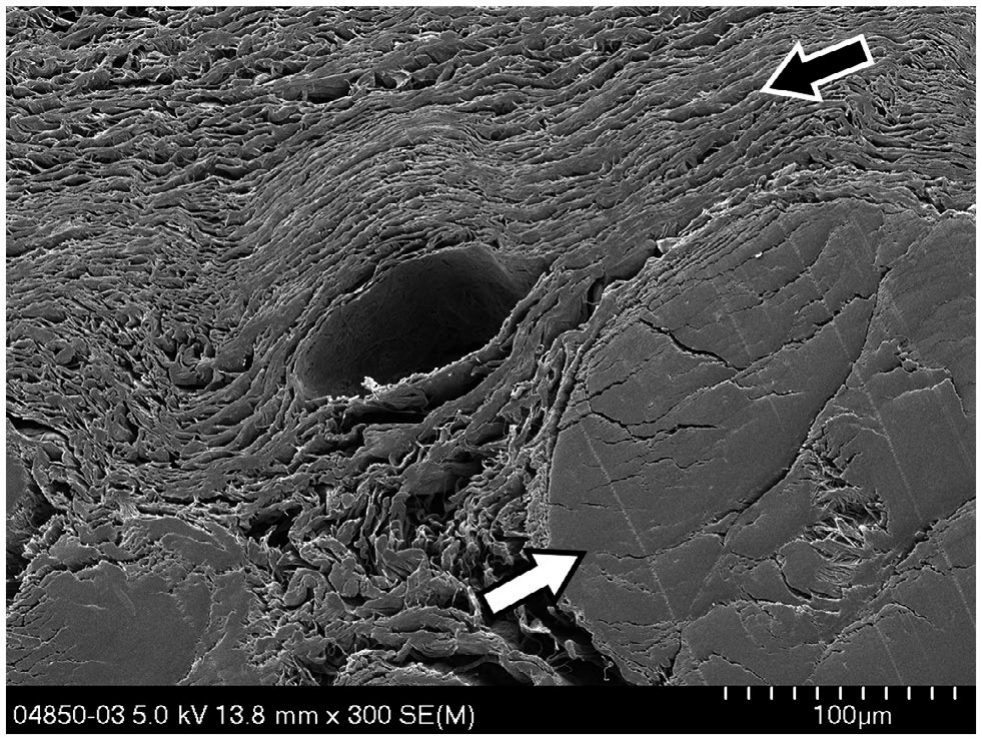
Transverse *SEM* view, x300, of a human superficial flexor tendon (white arrow) and the multilayered sheet organization of the SSCT (black arrow) as it is found throughout the carpal tunnel. A small vein present in the SSCT can be seen in the middle

The human CT consists of nine flexor tendons and the median nerve. These structures are enveloped by two synovial bursae and interconnected via a fibrovascular subsynovial connective tissue (SSCT). This connective tissue shows a unique arrangement in both humans as well as in animal models (Ettema, Amadio, Zhao, Wold, & An, 2004; Ettema, Zhao, An, & Amadio, 2006; Guimberteau, Delage, McGrouther, & Wong, 2010). Multiple horizontal sheets surround each tendon to form a gliding unit (Figure 1). The sheets are interconnected with smaller vertical fibrils that limit motion and prevent damage to the vascular and lymphatic structures (Guimberteau et al., 2010). This specific architecture also allows the absorption of mechanical stress as successive layers of SSCT are recruited sequentially with increased tendon excursion (Morizaki et al., 2012) preventing the nerve from excessive shear forces. The SSCT is hypothesized by some to play a central role in the pathophysiology of CTS (Ettema, Amadio, et al., 2006; Lluch, 1992; Tucci, Barbieri, & Freeland, 1997). Studies have shown that the SSCT is prone to irreversible damage even within physiological tendon excursion (Lluch, 1992; Morizaki et al., 2012). Under continued stress, the SSCT damage initiates a non‐inflammatory fibrotic response as is seen in histological samples of SSCT from CTS patients (Armstrong, Castelli, Evans, & Diaz‐Perez, 1984; Ettema et al., 2004; Ettema, Amadio, et al., 2006; Kerr, Sybert, & Albarracin, 1992; Lluch, 1992; Nakamichi & Tachibana, 1998; Oh et al., 2006; Phalen, 1966). Since movement and size of the SSCT can be measured non‐invasively, it is an interesting topic of investigation for diagnostic and prognostic purposes (Filius et al., 2015; Tat, Wilson, & Keir, 2015).

With the SSCT potentially playing a pivotal role in CTS development, a better understanding of its structural development over time is helpful. It is currently unknown whether the multilayer organizational structure of the SSCT develops purely in response to loading and motion over time or if it develops independent of stress loading during normal embryological tissue formation *in utero*. If the first case is true, it could be hypothesized that the SSCT starts as a simple structure that adapts over time due to finger, hand or wrist motion, similar to how bone and other tissue dynamically adapts to changes in force loading (Gillard, Merrilees, Bell‐Booth, Reilly, & Flint, 1977; Okuda, Gorski, An, & Amadio, 1987). This could, in turn, suggest that the SSCT damage in CTS is a failure of an adaptive mechanism, which might be addressed by physical measures; similar to the way stress fractures can be avoided by avoidance of sudden increases in repetitive loading of bone. However, if the SSCT is present in its adult form at birth, prior to high force dexterity loading, it would mean that the histological characteristics as seen in CTS patients are less of a defective adaptation response but more fitting with a progressive damage model. Potentially, a preset amount of fibrils are established during gestation which, during a lifetime of hand use, are gradually lost to attrition. In this case, there would be less of an indication to apply activity modification as a treatment, with strategies better targeting minimalization of the subsequent formation of SSCT fibrosis. The question would remain why some people develop CTS whereas others can go a lifetime without any problems; these two scenarios pose different possible solutions for the management of CTS.

With the difficulty in obtaining human fetal tissue, using an animal model showing a similar micro‐organization could help elucidate the development of the SSCT over time. A previous study described the comparability of the human SSCT to different animal models and concluded that the rabbit carpal tunnel was anatomically similar to humans (Ettema, Zhao, et al., 2006). This comparison study resulted in multiple CTS related rabbit studies (Chikenji et al., 2014; Morizaki et al., 2012; Yamaguchi et al., 2008; Yoshii, Zhao, et al., 2009; Yoshii, Zhao, Schmelzer, et al., 2011). Additionally, rabbit fetuses have been found to develop in utero in similar stages as humans (Beaudoin, Barbet, & Bargy, 2003). Therefore, the rabbit model was chosen to visualize the organizational structure of the SSCT before and after it has been subjected to physiological stress and strain of normal life. This article contains subjective descriptions of rabbit carpal tunnel morphology based on macroscopic, microscopic and ultramicroscopic characteristics, starting from third trimester fetuses to fully mature rabbits. These findings can be used to help design animal studies focused on carpal tunnel pathology. Emphasis will be placed on structural development of the SSCT sheets and the connections between.

## 2 MATERIALS AND METHODS

### 2.1 Animals

All animal protocols were approved by our institution's Animal Care and Use Committee (IACUC) and performed in accordance with the US National Research Council's Guide for the Care and Use of Laboratory Animals. Three male and three female adolescent (six weeks) New Zealand White (NZW; *Oryctolagus cuniculus L.*) and a pregnant NZW female at 27 days gestation were sedated intramuscularly with Ketamine (35 mg/kg) and Xylazine (5 mg/kg) and then euthanized with an intravenous overdose of pentobarbital; seven fetus kittens were collected from the pregnant female postmortem. Additionally, six 3‐day old rabbits and six 18–25 month old (four female and two male) NZW rabbit cadavers were donated from other IACUC approved studies from other laboratories; no interference between their research studies and our collected sample was expected. Sex in rabbits is not reliably visualized until 3–4 weeks of age therefore, sex was not noted for fetal and neonate groups. Immediately after sacrifice, both front legs were harvested at the point of the elbow and either frozen at −80°C or had the carpal tunnel excised and fixed according to imaging protocols below. Specimens were organized into the following age groups: fetal (27 day gestation; end of the third trimester), neonate (3 days), juvenile (6 weeks) and adult (18–25 months). To reduce the number of animals required, the group sizes were chosen for descriptive analyses only, and therefore objective quantification falls outside of the scope of this study.

### 2.2 Macroscopic features

Specimens were frozen at −80°C for at least 24 hr and cut with a diamond blade in transverse sections to acquire a full cross‐sectional view of the carpal tunnel for macroscopic imaging under magnifications ranging from x2‐10 (BLX51, Olympus, Japan). Macroscopic imaging of both fetal and neonate groups was not feasible due to limited tissue solidity and absence of discernable tissue characteristics at this age.

### 2.3 Microscopic features

Specimens underwent an *en bloc* style dissection of the carpal tunnel and flexor digitorum superficialis (FDS) of the third digit, taking care to not disrupt the SSCT. Sutures were placed at the proximal end for embedding orientation and to help maintain the integrity of the carpal tunnel. Samples were fixed in 4% neutral buffered formalin for at least 24 hr, dehydrated, cleared and subsequently paraffin‐embedded. Then, 5 μm sections cut both transversely and longitudinally were mounted on slides for standard hematoxylin and eosin (H&E) staining and evaluated under a maximum of x200 magnification.

### 2.4 Scanning electron microscopy features

Fresh samples from each age group were prepared for scanning electron microscope (*SEM*) imaging. The carpal tunnel was dissected *en bloc* as described above with a suture to indicate orientation the tissue was fixed in Trump's fixative as per standard protocol (McDowell & Trump, 1976) (1% glutaraldehyde, 4% formaldehyde and 0.1M phosphate buffer at pH = 7.2). Samples were then treated with a series of dehydration and rinse steps using, respectively, ethanol and 0.1 phosphate buffer in a critical point dryer. Cross‐sectional and longitudinal samples were mounted and sputter coated with gold‐palladium; images were taken with a cold field emission scanning microscope at 3.0kV (Hitachi S‐4–700, Hitachi High Technologies America, Inc., Pleasanton, CA, USA). In order to get both overview images as well as detailed organizational representations, magnifications between x80‐8.00k were taken for independent evaluation by two reviewers with subsequent consensus through discussion. Results were subsequently confirmed by two senior researchers who have published on this topic.

## 3 RESULTS

### 3.1 Gross overall anatomy – Human versus rabbit

**Figure 2:**
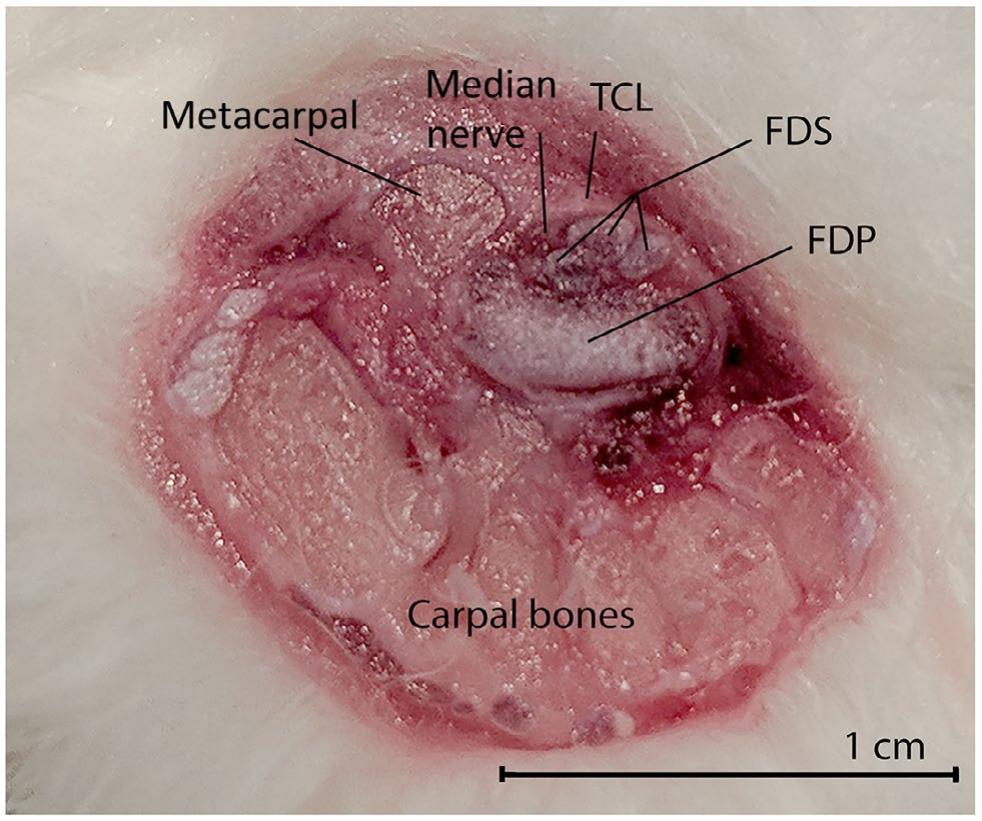
A macroscopic view of a transversely cut adult rabbit CT. Volar side at the top, radial side on the left of the image. There are three FDS tendons visible, superficial to the much larger singular FDP tendon. The median nerve is located just radial to the tendons

The adult rabbit carpal tunnel has been described in detail before, including similarities with human anatomy (Ettema, Zhao, et al., 2006). In short, both carpal tunnels contain digital flexor tendons, the median nerve, in some cases a (persistent) median artery, and a transverse carpal ligament covering the volar side. In adult rabbits, the carpal tunnel is about 8 mm proximal to distal compared to 21–30 mm in humans. The transverse ligament has two harder triangularly shaped fibrocartilageous areas on the lateral edges and a thinner section overlapping the CT. A marked difference with humans, however, is that the deep flexor tendon (FDP) in rabbits does not branch into the individual tendons proximal but distal to the carpal tunnel. Often, at the level of the CT, there are only three individual FDS tendons visible, of which one splits more distally into two. Additionally, the fifth metacarpal is located more proximally than in humans (dewclaw); the flexor pollicis longus lies dorsal to the FDP. The proximal carpal bones form the dorsolateral arch in which the CT lies. Rabbits have a total of nine carpal bones, with an extra central carpal bone in the distal row (Popesko, Rajitová, & Horák, 1992). As seen in Figure 2, the carpal tunnel lies volar to the carpal bones and measures 5–6 mm in width and about 4 mm in height. In one of the adult front paws, the nerve split into a ramus medialis and ulnaris just before the CT, as described earlier by Ettema, Zhao, et al. (2006). Based on our observations, the macroscopic CT layout of the juvenile rabbits was similar to that of the adult rabbits.

### 3.2 Histological assessment of rabbit samples

**Figure 3:**
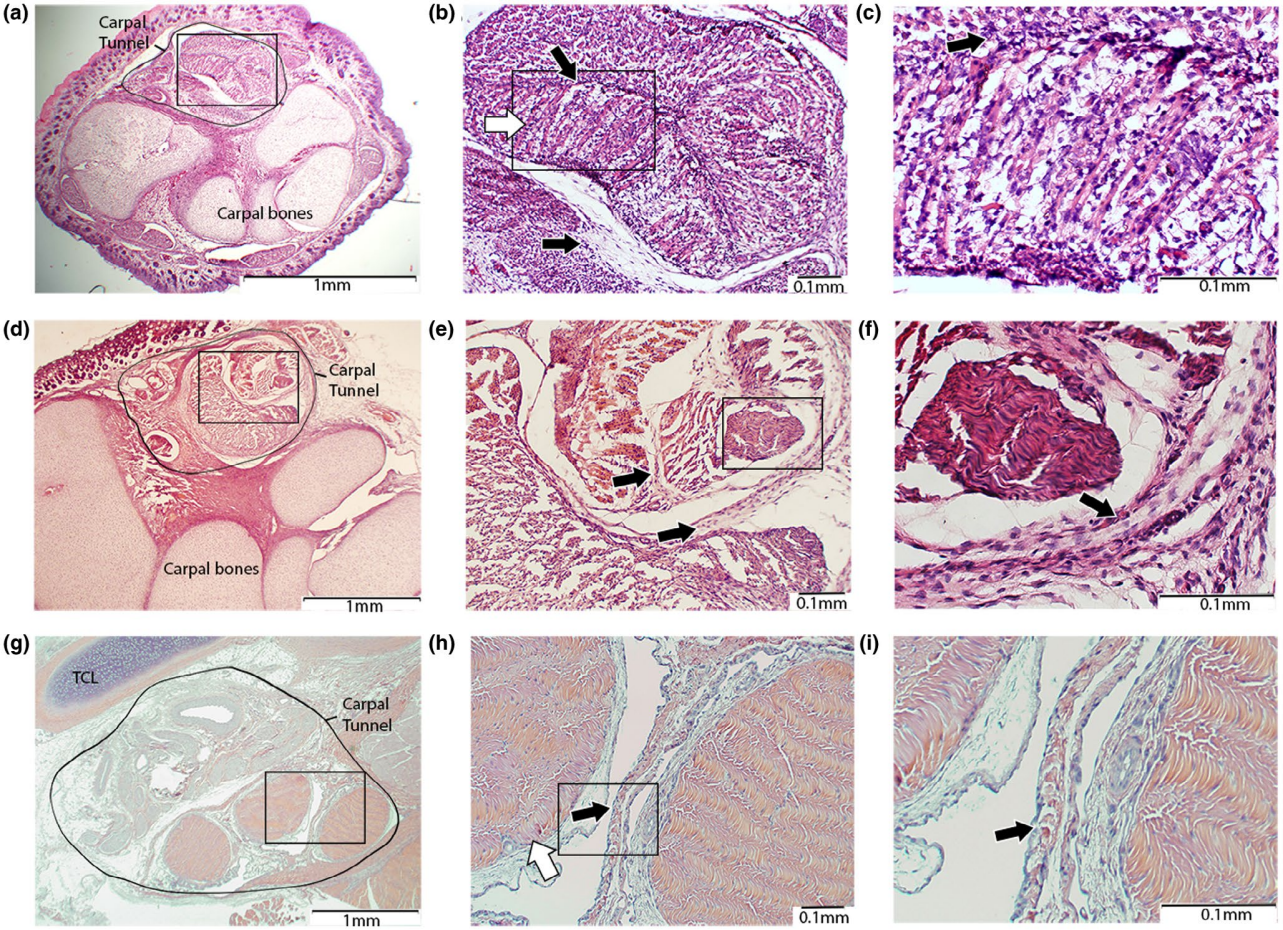
H&E staining of the CT of a rabbit at 27 days gestation (top row), 3 days post‐partum (middle row), 6 weeks post‐partum (lower row). (A–C) The carpal tunnel is recognizable including the deep flexor tendon and the three FDS tendons which. Connective cell layers within and around the tendons are seen. (D‐F) The tendons have separated, but are still closely related. The SSCT is better distinguishable and thicker around the circumference of the carpal tunnel compared to in between the tendons. (G‐I) The FDS tendons are fully isolated with a connective cell layer of just a few cells wide separating them. CT: carpal tunnel; FDS: flexor digitorum superficialis; TCL: transverse carpal ligament; SSCT: Subsynovial connective tissue. Rectangles: region of interest for subsequent pictures. Arrows: superficial flexor tendon (white), connective tissue (black). Magnifications of x20, x100 and x200 were used.

Histological assessment of the fetal tissue showed fully developed carpal bones and the completed formation of the isolated carpal tunnel (Figure 3a). The FDS tendons form a closely packed bundle superficial to the FDP. At higher magnification, the tendons display high cellularity with a small nuclei‐matrix ratio, fitting the highly proliferative fetal stage (Figure 3b, white arrow). A cellular lining of connective tissue was located circumferentially to the FDS bundle as well as in between the individual FDS tendons (Figure 3b‐c, black arrows). At 3 days post‐partum, the tendons showed a decrease in cellularity and were more clearly defined compared to the fetal samples. Where the fetal samples had more densely packed tendon bundles, the post‐partum samples showed more individualized tendons separated by a layer of connective cells. This connective layer between the individual FDS tendons was considerably smaller than the layer around the CT (Figure 3e–f). At 6 weeks, all the carpal tunnel structures including the median nerve and the transverse carpal ligament had fully formed, comparable to the adult stage. The SSCT was present throughout the CT, again with a thicker layer around the CT compared to the layers in between the tendons (Figure 3g–I).

### 3.3 Electron microscopic images of rabbit tissue

#### 3.3.1 Fetal (27 day gestation)

**Figure 4:**
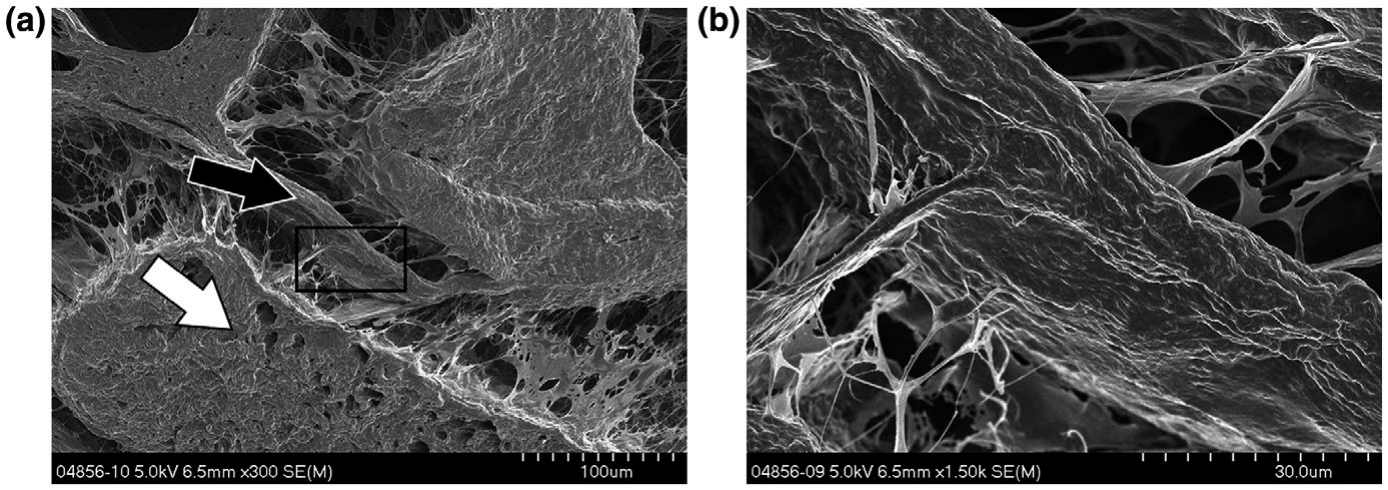
Transverse *SEM* views of the carpal tunnel structures of a third trimester rabbit kit. The FDP tendon has been formed (white arrow) and has a dense, homogenous matrix on top (black arrow). A higher magnification, broad sheeted fibres are connecting the tendon to the dense matrix

Working chronologically from young to old, the *SEM* images of fetal CT showed distinguishable oval shapes of the flexor tendons (Figure 4a), with a characteristic wave‐like organization pattern in the longitudinal view. Regions of SSCT matrix were mostly selected around the flexor tendons, assuming that changes in organizational structure would most likely be presented in the areas subjected to the highest shear stress levels. At higher magnification (Figure 4b), the surrounding connective tissue appeared as a dense, solid mass with sporadic connections to the tendon. These connections appeared as single‐stranded thin fibrils with flat surfaces and large footprints on the tendon. No difference in connective tissue thickness or organization was seen at other regions in the carpal tunnel.

#### 3.3.2 Neonate (3 days)

**Figure 5:**
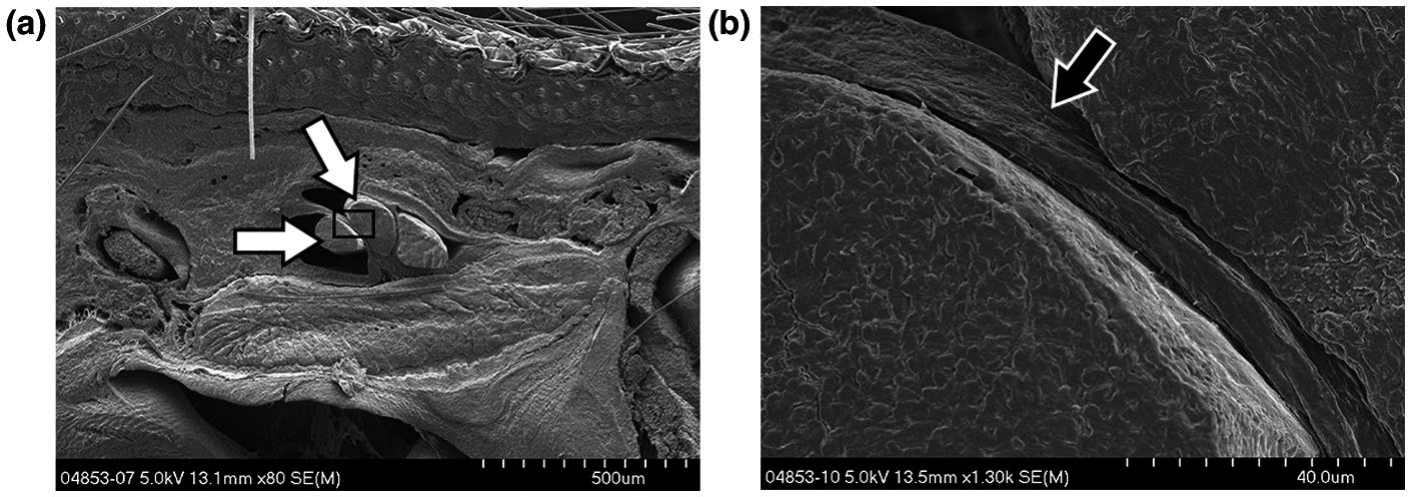
*SEM* images showing transverse carpal tunnel tissue from a 3 day old rabbit kit. (A) The FDS tendons are discernable (white arrows) and lie volar to the flattened FDP. The palmar side with the skin can be seen at the top. (B) A thin layer of dense tissue separates the flexor tendons (black arrow)

*SEM* images from neonates showed that the flexor tendons have clearly formed and are separated by a thick, dense layer of connective tissue, as was seen in the fetal tissue (Figure 5a). Fibrils looked similar in terms of morphology and gross count and were again represented as flat connectors, although not present throughout (Figure 5b). It is noteworthy that rabbits are not ambulating at this age, but will show active motion and gross limb movement.

#### 3.3.3 Juvenile & Adult (6wk and adult)

**Figure 6:**
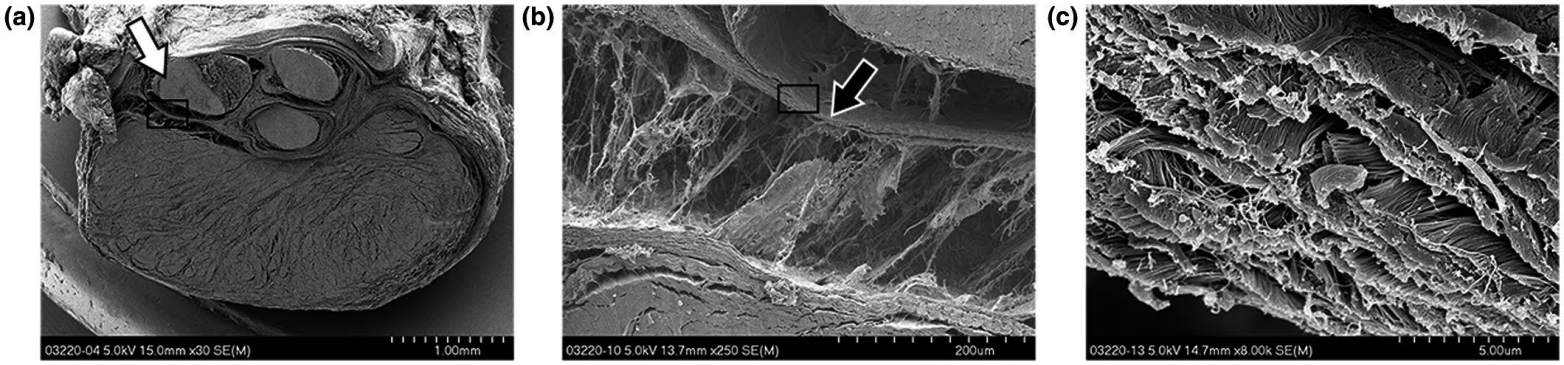
*SEM* images of a fully dissected carpal tunnel from a juvenile rabbit containing the FDP and three FDS tendons. Sample was cut transversely. (a) At low magnification, the individual FDS tendons (white arrow) are distinguishable with the much larger FDP bundle at the bottom. (b) In between the FDS and FDP, a thin layer of the SSCT is visible (black arrow), with loosely attached string‐like fibers attached to the FDP. (c) At higher magnification, the intricate sub‐organization of the sheet is visible, with flattened bundles of fibrils oriented parallel to the tendon

**Figure 7:**
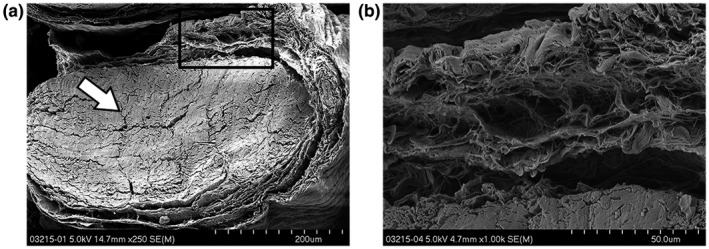
*SEM* images of a juvenile rabbit with (a) a single FDS tendon (white arrow) and the surrounding SSCT. (b) Initial layering starts superficial to the tendon with a network of crossing bundles and relatively thick sheets, partially merged together

**Figure 8:**
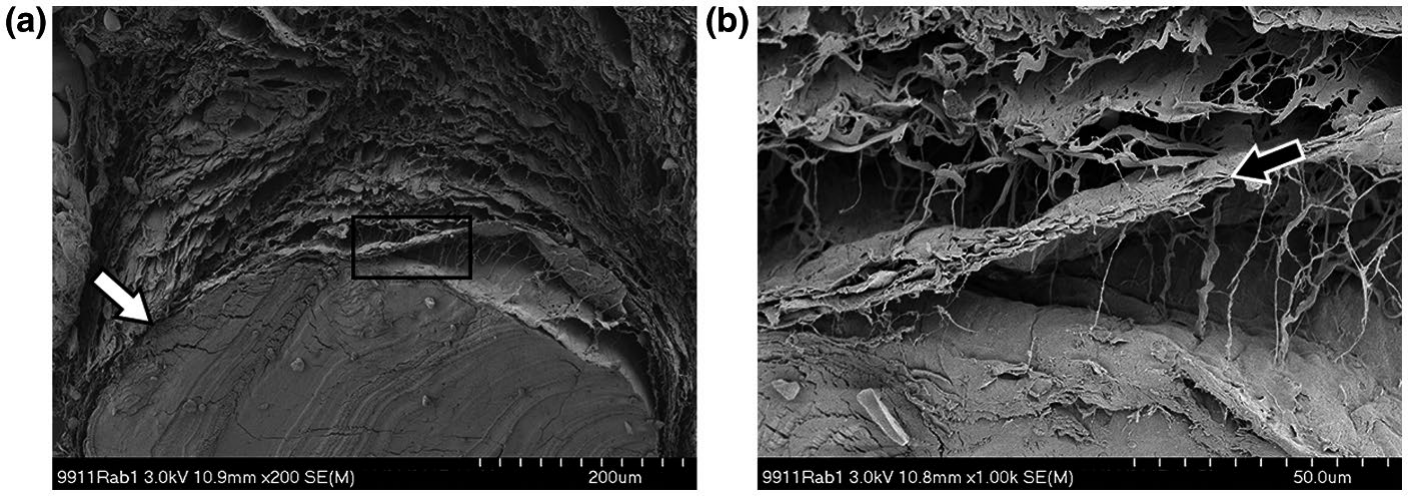
*SEM* image of adult rabbit carpal tunnel tissue, cut in the transverse plane. (a) The SSCT superficial to the third FDS tendon (white arrow) shows a structural organization similar to humans with multiple horizontal sheets (b) stacked on top of each other (black arrow) and linked through smaller singular fibrils

In these samples, a more profound difference in connective tissue morphology was visible. With the layered organization, the connective tissue at the juvenile age (Figure 6a&b) more closely resembles what has previously been described in the adult (and human) SSCT. Instead of the densely packed homogenously appearing matrix, sheets now have formed, giving the transverse cross‐section a more layered and separated appearance. However, the presence of the interconnecting fibrils is less apparent at the juvenile stage (Figure 6c) than in the adult. At an x8.0k magnification, the transverse cross‐sectioned sheets appear to be the product of flattened bundles of connective matrix, not interconnected but still closely stacked. In some of the samples, the sheet formation was not as profound and seemed more chaotic and interwoven (Figure 7). However, none of the images showed the solidity of the SSCT as seen in the younger specimens. This was true for the SSCT between the FDS tendons as well as around individual tendons. The adult specimens had a more layered appearance with a higher count of sheets and larger spaces separating them (Figure 8a). The original orientation of the fibrils within the sheets was still visible although the surface appeared smoother (Figure 8b).

## 4 DISCUSSION

Between the final fetal stage and adulthood, the connective tissue in a rabbit carpal tunnel undergoes marked morphological changes, from a dense, thick matrix localized peri‐ and inter‐tendinous to a complex micro‐organization of interlinked sheets comprised of flattened collagenous fibrils. Assuming that there is a reasonable comparison to make between rabbit and human development (Beaudoin et al., 2003), looking at the rise and timing of the shaping of the SSCT supports the adaptive development hypothesis described in the introduction, which may lead to important implications regarding the development and possibly even management of carpal tunnel syndrome.

One of the most interesting findings was the transition from a solid structure into the multilayered one between the fetal and neonatal time points. As in tendon and ligament development, it seems plausible that SSCT development is intimately connected with the development of its anatomical context and the application of mechanical forces (Wortham, 1948). Tendons have been shown to react in cellular response to mechanical forces and increases in shear stress, adjusting the type of extracellular matrix deposited (Gillard et al., 1977). Animals that need independent locomotor movement quickly after birth show larger tendon fibril diameters than those who can remain inactive after birth (Parry, Craig, & Barnes, 1978). Although our study lacks time points between 3 days and six weeks, it is not until after the first week that rabbits will start exhibiting controlled active movement and will start bearing their weight on the front paws (Mills & Daniel, 1993), so it seems fitting that it was not until the latter time point that we found marked differences in SSCT structural organization. This is supported by an earlier study that looked at rabbit superficial flexor tendon and synovial sheath development, which noted that it was not until after the first month that larger tendon fibrils with more interconnections were formed (Oryan & Shoushtari, 2008). The authors also described that the synovial membrane surrounding the tendon showed less mature fibroblasts compared to the tendon proper up to 112 days after birth. Although this sampling was done at a level more proximal than the CT and only reviewed tissue on microscopic level, this indicates that the connective tissue lags in development and, as the authors suggest, could be due to a response to direct loading of the tendon whereas the sheath is subjected to less and indirect loads (Chaplin & Greenlee, 1975; Oryan & Shoushtari, 2008). Additionally shown was the tendon decreasing in cellularity to 5% of the original count within the first 4 months of life, which was reflected in our histological observations as well.

In order to link our anatomical findings to a functional purpose, we hypothesize that nutrition transportation and mechanical stress inhibition play prominent roles. First, it is important to note that flexor tendons in the carpal tunnel are unique in that they do not conform to the conventional categories of being either intra‐, of extrasynovial. The loose paratenon (or epitendineum) commonly found lining the outer layer of a tendon provides nutrition for extrasynovial tendons (Gelberman, Seiler, Rosenberg, Heyman, & Amiel, 1992), but this layer is incorporated in the SSCT at the level of the carpal tunnel. This combination of a synovial lubrication layer and the viscoelastic character of the SSCT allows for enhanced movement with less friction (Zhao et al., 2007). Guimberteau et al., (2010) describe the SSCT as a microvacuolar network containing blood and lymphatic vessels, showing highly variable polyhedral shapes when dissected. Chemical analysis of the SSCT showed a composition of predominantly proteoglycans (70%) and collagen type I and III (23%) which fits with the relatively high water content and the mucinous‐like nature noticeable *a vue.* Secondly, if the main function of the connective tissue surrounding the flexor tendons can be summarized as (1) modulation of the transmission of stress (either in the form of shear or compressive) from the tendon to the surrounding tissue and (2) accommodating a vasculature network, then it would make sense that the SSCT structure is a combination of lubricant holding (i.e., the proteoglycans) and sliding (the SSCT layers) elements. The smaller interconnecting fibrils restrict movement, reducing the risk of damaging the nutritional sources (Guimberteau et al., 2010). When expanded, the SSCT provides an increase in maximal excursion by recruiting different layers, but is restricted when the edges reach the physical limitations of the preceding layer. This is comparable to extending a telescope. A multilayered system allows both the potential to capture fluid in between the layers and dampen stress with tendon loading (Filius et al., 2015; Yoshii, Villarraga, et al., 2009). Cadaveric and clinical data has shown that the SSCT moves less compared to the tendon during finger flexion and extension and. Their ratio depends on tendon velocity, total excursion and acquisition method (Schrier, Evers, Bosch, Selles, & Amadio, 2019; Yoshii, Zhao, Henderson, et al., 2011), with a stress‐relaxation pattern suggesting visco‐elastic behaviour (Morizaki et al., 2012). This would fit the above mentioned mechanism of shearing layers with incremental recruitment. Placing this in the context of CTS, it has already been shown that there is thickening and tearing of these fibrils in patients with CTS (Donato et al., 2009; Ettema, Amadio, et al., 2006) which lead to the hypothesis that SSCT damage could play a significant role in CTS pathophysiology (Festen‐Schrier & Amadio, 2018).

Assuming that one of its primary functions is to create a dynamically accommodating environment, it makes sense to look at a similar example, e.g. the muscle‐fascia interface; in humans, fetal movement commences as early as seven weeks of gestation with individual limb motion around week ten (Verbruggen et al., 2016). A study on myofascial development in older human fetal tissue (25–33 weeks) shows a cyclic process of fascia depositions, originating from the skeletal muscles themselves, then thickening, and subsequently detaching. The repetition of the process produced multilayered fascia (Cho et al., 2018). Hypothetically, a similar process could be present in the CT, where tendinous fibroblasts create layers of collagen matrix deposits in areas where friction is present (closest to the tendon), which would explain why the SSCT in the juvenile rabbits contained fewer layers and interconnecting fibrils compared to the adult rabbits.

A limitation of our approach is the absence of time points between 3 days and 6 weeks and the lack of biomechanical testing data. In humans, the SSCT does not exceed 1mm in width and has proven to be very difficult to reliably test biomechanically, especially in vivo. Based on our experience in this field, a preliminary observational study was deemed more feasible and so we wanted to limit the animal sample size. Most of our specimens were provided through other research studies, and the fetal and juvenile time points were chosen based on expected tendon development as reported by others. Nonetheless, these limitations prevent us from drawing any conclusions on causality and on how the SSCT is formed on a cellular level. As we wanted to appreciate not only SSCT development, but also the relationship between the different carpal tunnel structures during development and gain some insights into levels of cellularity, we choose to visualize the specimens in multiple ways. Unfortunately, due to limited tissue size and rigidity, we were not able to acquire the macroscopic images of the fetal and neonatal groups. Additionally, a few side notes need to be addressed concerning the limitations of our methodological approach. For the staining and *SEM* acquisitions, tissue was prepared through dehydration and rinse cycles. We cannot exclude the possibility that the dehydration might have affected some of the structural integrity of the specimens. Additionally, for both approaches samples needed to be cut, which in some samples resulted in minor dissociation of tendinous structures from surrounding connective tissue. SSCT tissue was mostly spared, despite this being quite delicate, probably because shear stress was limited through the addition of anchoring sutures on the edges of each sample. Finally, because of the selected method of specimen collection and the limited amount of tissue available per specimen, we were not able to fully correlate tissue characteristics between different visualization methods within one animal. Future research can build on our findings and further explore the developmental potential of a rabbit carpal tunnel model while answering clinically important questions. A follow‐up research question would be to test the effect of short‐term casting on SSCT morphology and strength, similar to what we do in conservative CTS treatment.

To summarize, the SSCT in the carpal tunnel is a unique structure with an important role in load transmission, which may be relevant in CTS pathology. Using an animal model, we have shown that, as in other musculoskeletal structures, the SSCT does not fully develop during the fetal phase but is instead more likely a functional adaptation to (post‐natal) activity. We propose that the structural layout of the SSCT supports minimization of load‐induced mechanical stress while facilitating nutritional flow, but further biomechanical studies are needed to confirm this hypothesis. We hope that a better understanding of the SSCT multi‐level organization will be useful to those who study the development of CTS, as well as those interested in CTS treatment.

## CONFLICT OF INTEREST

All the authors declare no conflicts of interest with the work presented here.

## AUTHOR CONTRIBUTION

**Verena Schrier:** Conceptualization; Data curation; Formal analysis; Investigation; Methodology; Writing‐original draft; Writing‐review & editing. **Alyssa Vrieze:** Data curation; Formal analysis; Investigation; Writing‐original draft; Writing‐review & editing. **Peter C. Amadio:** Conceptualization; Methodology; Resources; Supervision; Writing‐review & editing.
